# Exposure to heat at work: development of a quantitative European job exposure matrix (heat JEM)

**DOI:** 10.5271/sjweh.4243

**Published:** 2026-01-01

**Authors:** Tosca OE de Crom, Bernice Scholten, Eugenio Traini, Koen van der Sanden, Boris Kingma, Floris Pekel, Manosij Ghosh, Hilde Notø, Michelle C Turner, Miguel Angel Alba Hidalgo, Lisa Klous, Maria Albin, Henrik A Kolstad, Jenny Selander, Calvin Ge, Anjoeka Pronk

**Affiliations:** 1 Department of Risk Analysis for Prevention, Innovation & Development, Netherlands Organization for Applied Scientific Research TNO, Utrecht, The Netherlands.; 2 Department of Human Performance, Netherlands Organization for Applied Scientific Research TNO, Soesterberg, The Netherlands.; 3 Section for Integrative Physiology, Department of Nutrition, Exercise and Sport, University of Copenhagen, Copenhagen, Denmark.; 4 Department of Public Health and Primary Care, Centre for Environment and Health, Katholieke Universiteit, Leuven, Belgium.; 5 National Institute of Occupational Health (STAMI), Oslo, Norway.; 6 Barcelona Institute for Global Health (ISGlobal), Barcelona, Spain.; 7 Universitat Pompeu Fabra (UPF), Barcelona, Spain.; 8 CIBER Epidemiología y Salud Pública (CIBERESP), Madrid, Spain.; 9 Department of Occupational and Environmental Medicine, Lund University Hospital, Lund, Sweden.; 10 Institute of Environmental Medicine, Karolinska Institutet, Stockholm, Sweden.; 11 Department of Occupational Medicine, Danish Ramazzini Centre, Aarhus University, Aarhus, Denmark.

**Keywords:** epidemiology, exposure assessment, heat stress, occupational exposure, wet bulb globe temperature

## Abstract

**Objective:**

With climate change exacerbating occupational heat stress, objective and systematic exposure assessment is essential for epidemiological studies. We developed a job exposure matrix (JEM) to assign occupational heat stress exposure across Europe.

**Methods:**

Aligned with the International Organization for Standardization (ISO: 7243, 8996 and 9920), the heat JEM provides region- and year-specific estimates of annual heat stress hours by job title, using the International Standard Classification of Occupations 1988 for Europe [ISCO-88(COM)]. Heat stress was defined as wet bulb globe temperature effective (WBGT_eff_) exceeding WBGT reference (WBGT_ref_). Outdoor and indoor WBGT were determined using historical, region-specific hourly meteorological data (temperature, radiation, humidity, wind speed) across Europe, between 1970 and 2024. WBGT values were adjusted for job-specific clothing to obtain WBGT_eff_. WBGT_ref_ was based on metabolic rate, calculated using body surface area and job-specific physical activity, and adjusted for acclimatization status. Further adjustments were made for the job title-specific presence of local heat and cooling sources, time spent indoors versus outdoors, and working schedules.

**Results:**

The number of annual hours workers experience heat stress is highest among jobs involving local heat sources and physical demanding tasks, especially when work clothing is mandatory. Southern Europe has a higher annual heat stress burden compared to other regions. Exposure varies across calendar years and is substantially higher among unacclimatized versus acclimatized workers.

**Conclusions:**

Incorporating job-, region-, and year-specific factors, the heat JEM provides a harmonized tool for studying occupational heat stress. Its transparent framework allows for updates with new data and extensions to other years and regions.

The International Labour Organization has reported that at least 2.41 billion workers worldwide are exposed to heat stress annually (1), and this number is expected to increase due to climate change. The risk of heat stress is particularly high in outdoor jobs that involve physically demanding tasks and require work clothing (2, 3). Indoor workers exposed to local heat sources, such as laundering and baking, are also at a high risk of heat stress (4, 5).

Exposure to heat stress poses serious health risks, ranging from heat exhaustion and heat stroke to death (6–8). Beyond these acute effects, prolonged heat stress may also cause chronic conditions, such as cardiovascular disease (9), kidney damage (10), and mental health disorders (11). Although these risks are well recognized and workers are known to be particularly vulnerable, data on the specific health effects of occupational heat stress remain limited. Most studies on this topic focus on the acute impacts and often rely on relatively small field studies conducted in tropical climates (12, 13). Consequently, the long-term effects of occupational heat stress, especially under more moderate climate conditions and among susceptible subgroups, remain insufficiently understood. To address these knowledge gaps, large-scale epidemiological studies assessing the long-term health effects of occupational heat stress across diverse climatic regions are necessary, which in turn requires tools for heat stress estimations.

Job exposure matrices (JEM), which define exposures based on job titles, are commonly used tools to evaluate standardized occupational exposures in epidemiological studies systematically (14). To reliably assess occupational heat stress, a heat JEM must account for job-specific factors, including physical activity levels that increase the metabolic rate (15), mandatory clothing that limits heat dissipation (16), the presence of local heat and cooling sources, and the proportion of time spent indoors versus outdoors. Accurate assessment also requires accounting for time-varying regional meteorological factors, including air temperature, humidity, air movement, and thermal radiation (17). The wet bulb globe temperature (WBGT) index offers an established framework for integrating these elements, allowing for comprehensive, job- and region-specific heat stress estimates over time (18). Additional modifiers, such as body surface area, acclimatization status (19), and work organizational factors (eg, working hours and vacation days), can further influence heat stress but are often unavailable in epidemiological datasets. Incorporating these factors where possible improves exposure accuracy, which highlights the need for a transparent, adaptable framework for a heat JEM.

A Finnish heat JEM was previously developed (20), and a local expert panel of five industrial hygienists later adapted it to Spanish working conditions (21). In alignment with international standards, occupational heat exposure in this JEM was defined as “heat from natural or artificial sources continuously exceeding threshold levels of the WBGT index” (22). However, this JEM lacks details on how job-specific factors and regional variations in WBGT parameters are incorporated, which limits its adaptability. As such, a more comprehensive and adaptable tool is needed to assess occupational heat stress across diverse settings.

This study aimed to develop a JEM to estimate annual occupational heat stress across Europe from 1970 to 2024, incorporating job-, region-, and year-specific variations. The heat JEM follows a transparent framework, allowing for future updates and customized applications in epidemiological studies.

## Methods

### Conceptual framework and theoretical basis

The heat JEM provides region- and year-specific estimates of the total annual working hours under heat stress, assigned to the 4-digit job codes derived from the International Standard Classification of Occupations 1988 for Europe [ISCO-88(COM)]. The JEM was based on the heat stress assessment standards set by the International Organization for Standardization (ISO). Specifically, we referred to ISO 7243 (23), which offers guidance on estimating occupational heat stress using the WBGT index; ISO 8996 (15), which provides methods for determining the metabolic rate during various work activities; and ISO 9920 (16), which outlines procedures for estimating the thermal characteristics of clothing. In addition, we built further on a framework developed to characterize occupational heat exposure within the HEAT-SHIELD project (17). In this context, heat stress is defined as occurring when the estimated WBGT effective (WBGT_eff_) exceeds the WBGT reference (WBGT_ref_). WBGT_eff_ is a measure of environmental heat experienced by workers adjusting for the effect of clothing, while WBGT_ref_ reflects the tolerance thresholds for heat stress considering metabolic rate and acclimatization status.

**Figure 1:**
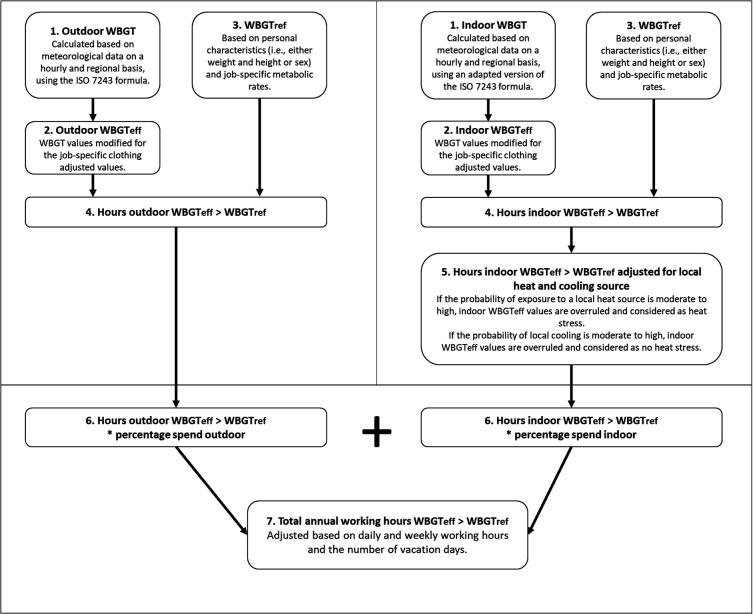
Framework underlying the heat job exposure matrix (JEM) based on wet bulb globe temperature (WBGT). The framework outlines the determination of total annual working hours of heat stress by assessing whether effective wet bulb globe temperature values (WBGTeff) exceed reference values (WBGTref), according to standards set by the International Organization for Standardization (ISO).

Figure 1 illustrates the stepwise framework underlying the heat JEM. Throughout the framework, several job-specific input parameters are introduced, including occupational physical activity, whether work tasks are typically performed indoors or outdoors, mandatory work clothing and head cover, and the presence of local heat or cooling sources. A detailed description of each step and the assessment of input parameters is provided in the supplementary material (www.sjweh.fi/article/4243), and a brief summary is included below.

The heat JEM algorithm was written in R statistical software version 4.3.2.

### Assessment of input parameters

Occupational physical activity and the ratio of time workers spent indoors versus outdoors were derived from pre-existing JEM, as described in detail elsewhere (23, 24). Information on all other parameters was obtained by expert judgment assessments, in some cases guided by a literature search. These expert judgements were conducted by five experts from four different countries, including the Netherlands (LK, CG), Belgium (JB), Spain (MAAH), and Norway (HN), with the initials referring to the contributing coauthors. The group included specialists in occupational hygiene (CG, HN, JB, MAAH), occupational exposure assessment (CG, HN, JB, MAAH), and thermal physiology (LK). The ratings were performed independently and discordant ratings were discussed to reach consensus ratings. The final decisions made by the experts are provided in the supplementary material.

### Stepwise construction of the framework

*Step 1. Outdoor and indoor WBGT.* Outdoor and indoor WBGT values in degrees Celsius (°C) were calculated separately based on the fifth-generation reanalysis land (ERA5-Land) meteorological data derived from the European Centre for Medium Range Weather Forecasts (ECMWF) (25), using Phython statistical software. The meteorological data used for these calculations include hourly measurements of air temperature, relative humidity, wind speed, and radiant heat across Europe, at a 0.1° × 0.1° latitude-longitude resolution (approximately 9 × 9 km), covering the period from 1970 to 2024. Using this data, the natural wet-bulb temperature (tnw) and globe temperature (tg) were estimated using the Liljegren method (26). These estimates, along with the air temperature (ta), were used to calculate outdoor WBGT using the standard ISO 7243 formula:

Outdoor WBGT = 0.7 × t_nw_ + 0.2 × t_g_ + 0.1 × t_a_

Indoor WBGT was calculated assuming that radiant temperature equals dry bulb temperature, wind speed is 0.4 m/s, and short-wave irradiance is zero for the natural wet-bulb temperature calculation, which simplifies the formula to:

Indoor WBGT = 0.7 × t_nw_ + 0.3 × t_a_

To enable regional comparisons and align with administrative boundaries, WBGT values were aggregated to the Nomenclature of Territorial Units for Statistics (NUTS) levels 0, 1, 2, and 3. The NUTS classification is a hierarchical system from Eurostat for regional statistics in the EU, with NUTS 0 representing countries, NUTS 1 major regions, NUTS 2 policy-relevant areas, and NUTS 3 small administrative units such as provinces or departments. Any of these levels can be used in heat stress calculations, depending on the research context and the spatial resolution that best aligns with the geographic variability and objectives of the analysis. Grid cell values were assigned to NUTS regions based on the location of each cell’s centroid. For each NUTS level, the mean of all grid cell values within the region was then calculated to produce an aggregated NUTS estimate.

*Step 2. Outdoor and indoor WBGT_eff._* The outdoor and indoor WBGT values obtained in step 1 were modified by job-specific clothing adjustment values (CAV) to obtain the WBGT_eff_. The CAV were assigned to each ISCO-88(COM) job code by the expert panel based on mandatory work clothing and head covering. Jobs were categorized into three mandatory work clothing levels (low, medium, high), corresponding to +0, +3, and +11°C-WBGT, respectively. An additional +1°C-WBGT was added for mandatory head covering. Supplementary table S2 provides a full overview of the mandatory work clothing and head covering classifications, and their corresponding CAV.

*Step 3. WBGT_ref._* WBGT_ref_ was calculated based on the metabolic rate specific to each job, and the acclimatization status (15). The calculations were performed using the following formulas:

Unacclimatized workers: WBGT_ref_ = 59.9 – 14.1 × log_10_(metabolic rate)

Acclimatized workers: WBGT_ref_ = 59.9 – 11.5 × log_10_(metabolic rate)

Metabolic rate (in watts) was estimated using job-specific physical activity levels expressed as metabolic equivalents of task (MET, 1 MET ~ 58.2 watts per m^-2^) (15), derived from a previously developed JEM (23). The following formula was applied:

Metabolic rate = MET value × body surface area × 58.2,

where body surface area in m^2^ was estimated using the Du Bois formula (27):

Body surface area = 0.007184 × weight (kg)^0.425^ × height (cm)^0.725^.

In the absence of weight and height data, typical sex-specific body surface area values can be used (1.9 m^2^ for men, 1.6 m^2^ for women), acknowledging some population variability (28, 29). This assumes lower heat stress vulnerability in women due to their smaller average body surface area. No further sex-specific adjustments to the WBGT_ref_ were made, as evidence on sex-based heat vulnerability is inconsistent and no standard correction factors exist (30).

*Step 4. Heat stress hours classification (WBGT_eff_ >WBGT_ref_).* After obtaining WBGT_eff_ for each hour and WBGT_ref_ as a standard across all hours, heat stress was determined for each outdoor and indoor hour by evaluating whether WBGT_eff_ exceeds WBGT_ref_ (22).

*Step 5. Indoor heat stress hours adjusted for local heat and cooling sources.* For indoor settings, WBGT_eff_ values were further adjusted based on the presence of local heat or cooling sources, as assessed by the expert panel. During the hours workers were exposed to a local heat source while performing indoor tasks, the indoor WBGT_eff_ was overruled, and these exposure hours were classified as heat stress. Conversely, in the presence of local cooling, it was assumed that the indoor environment was sufficiently cooled below WBGT_ref_, resulting in no exposure to heat stress. Under this assumption, local cooling overrules the effect of the local heat sources.

*Step 6. Heat stress hours adjusted for outdoor and indoor work.* Total annual hours in which WBGT_eff_ exceeded WBGT_ref_ were first calculated separately for indoor and outdoor conditions (step 1–5 and 1–4, respectively). These hours were then weighted by the estimated job-specific proportion of working hours spent indoors versus outdoors, derived from an expert assessments of a prior UV exposure JEM (24).

*Step 7. Total heat stress hours adjusted for work organization factors.* Final annual hours in which WBGT_eff_ exceeded WBGT_ref_ were adjusted for work organization factors including daily and weekly working hours and vacation days. This can be tailored by cohort-specific norms.

### Application and illustrative analysis

This paper demonstrates analyses of modelled annual heat stress hour estimates generated by the Heat JEM. For each unique combination of job, year, region, acclimatization status, body surface area, and work organizational factors, the JEM produces a single deterministic estimate of heat stress hours. Estimates do not include within-cell variability or sampling error.

Calculations of all illustrative analyses were based on a reference scenario assuming unacclimatized workers with a body surface area of 1.9 m^2^ (ie, the average body surface area for men) in the calendar year 2020, assuming full-time employment from 09:00–17:00 hours (8 hours/day, 5 days/week) with 30 vacation days (230 workdays/year), unless stated otherwise. The average body surface area for men was chosen as men predominantly perform most jobs with a high risk of heat stress. Keeping all parameters constant allows for a clear indication of how job-specific factors influence heat stress.

We first determined the number of hours that workers experience heat stress annually for each of the 37 European countries (ie, at NUTS 0 level) and each ISCO-88(COM) job code, classified at the unit group level (ie, 4-digits).

To explore regional differences, we mapped heat stress exposure at NUTS 3 level for four jobs representing diverse exposure scenarios: “medical doctors” (ISCO 2221; mostly indoor with local cooling and low metabolic rate), “crop and animal producers” (ISCO 6130; mostly outdoor, moderate-to-high metabolic rate), “painters and related workers” (ISCO 7141; mixed indoor/outdoor, moderate metabolic rate), and “building construction laborers” (ISCO 9313; fully outdoor, high metabolic rate, with mandatory helmet use). These jobs were selected because they have well-defined work patterns, making heat stress estimates more reliable than for jobs with variable and unpredictable duties such as firefighters or armed forces.

We then examined temporal trends in annual occupational heat stress hours from 1970 to 2024 for the same jobs. Calculations were performed for Finland, France, Italy, Poland, and the United Kingdom, representing a broad range of European climates.

Finally, we performed sensitivity analyses to examine how heat stress exposure changed when input parameters were altered while keeping all other factors constant. Three variations to the reference scenario were examined (i): reducing body surface area from 1.9 m^2^ (average for men) to 1.6 m^2^ (average for women) (ii), changing working hours from full-time (09:00–17:00 hours) to siesta time (08:00–12:00 hours and 16:00–20:00 hours), and (iii) changing from unacclimatized to acclimatized workers. Analyses were performed for “crop and animal producers” (ISCO 6130) and “building construction laborers” (ISCO 9313) in France, Italy, and Poland. These jobs and countries were selected to reflect diverse work environments and climates, with sufficient exposure variation to assess the parameter effects meaningfully.

## Results

Supplementary tables S4a–d present how the calculations are performed step-by-step through the seven steps of the heat JEM framework for “medical doctors” (ISCO 2221), “crop and animal producers” (ISCO 6130), “painters and related workers” (ISCO 7141), and “building construction laborers” (ISCO 9313).

### Heat stress exposure across jobs and regions

**Figure 2:**
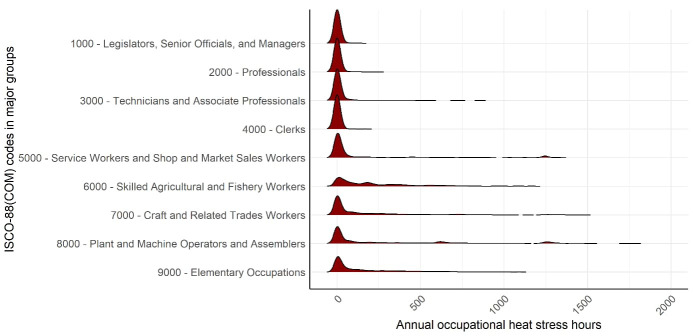
Variation in annual occupational heat stress hours across major groups of jobs in European countries. Each ridge represents the variation in annual occupational heat stress hours across 37 European countries for the corresponding ISCO-88(COM) codes in major groups listed on the y-axis. Estimates were calculated at the 4-digit ISCO-88(COM) code level but are presented here grouped by the nine major ISCO-88(COM) categories for visualization purposes. Higher peaks indicate that most workers within an job experience similar heat stress hours across regions, whereas wider distributions reflect greater variation in heat stress hours across regions within that job. Calculations are based on unacclimatized workers with a body surface area of 1.9 m^2^ (average for men) in 2020, assuming full-time employment from 09:00–17:00 hours (8 hours/day, 5 days/week) and 30 vacation days (230 workdays/year).

Figure 2 shows the distribution of occupational heat stress hours in 2020 across jobs and the 37 European countries (NUTS 0). Results are grouped by the nine major groups of the ISCO-88(COM) job codes, although the estimates are based on analyses conducted at the 4-digit level of individual ISCO-88(COM) codes. Jobs classified under ISCO-88(COM) codes starting with 1 (legislators, senior officials, and managers), 2 (professionals), 3 (technicians and associate professionals), and 4 (clerks) generally experience relatively few occupational heat stress hours. For these jobs, heat stress exposure ranged from 0–450 hours annually across different jobs and countries, with exceptions for “athletes, sports persons and related associate professionals” (ISCO 3475, ≤732 hours) and “ships’ engineers” (ISCO 3141, ≤858 hours). More variability, with a skew toward higher annual heat stress hours, was observed in jobs classified under ISCO-88(COM) codes starting with 5 (service workers and shop and market sales workers), 6 (skilled agricultural and fishery workers), 7 (craft and related trades workers), 8 (plant and machine operators and assemblers), and 9 (elementary occupations). For these jobs, annual heat stress exposure varied by job and country, ranging from 0–1788 hours out of a maximum possible 1840 hours per year (calculated as 230 workdays × 8 hours per day).

Supplementary figure S1 provides the distribution of heat stress hours in 2020 across the 37 European countries (NUTS 0) for all ISCO-88(COM) coded jobs. Seven jobs were affected by a local heat source for 6.5 hours per day, which consistently caused high heat stress levels with little variation across regions, ranging from 1245–1484 hours annually. These jobs include “cooks” (ISCO 5122), “metal moulders and coremakers” (ISCO 7211), “ore and metal furnace operators” (ISCO 8121), “metal melters, casters and rolling-mill operators” (ISCO 8122), “metal heat-treating-plant operators” (ISCO 8123), “glass and ceramics kiln and related machine operators” (ISCO 8131), and “glass, ceramics and related plant operators not elsewhere classified” (ISCO 8139). The top three jobs with the greatest variation in annual heat stress hours across regions, ranging from 0–1788 hours per year, include “inland and coastal waters fishery workers” (ISCO 6152), “deep-sea fishery workers” (ISCO 6153), and “motorcycle drivers” (ISCO 8321). For the first two jobs (ISCO 6152 and 6153), variation was mainly be due to high physical demands (4.55 MET and 3.98 MET, respectively), and added clothing burden (CAV +4°C). For motorcycle drivers, clothing was the main driver (CAV +11°C), with physical demands contributing to a lesser extent (3.06 MET).

**Figure 3:**
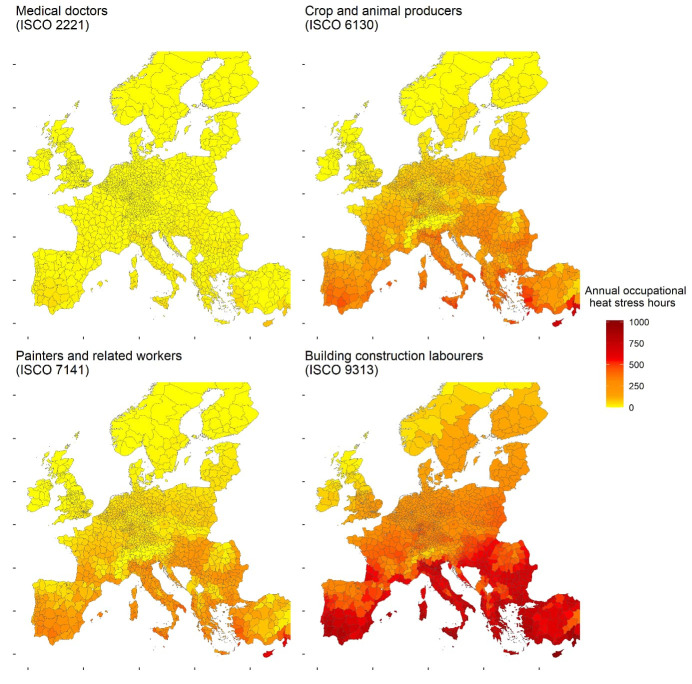
Annual occupational heat stress hours across Europe for selected jobs in 2020. Calculations are based on unacclimatized workers with a body surface area of 1.9 m^2^ in 2020, assuming full-time employment from 09:00–17:00 hours (8 hours/day, 5 days/week) and 30 vacation days (230 workdays/year).

Figure 3 presents maps of annual heat stress exposure hours across regions in Europe for four selected jobs. Exposure is consistently higher in southern regions, with Cyprus showing the highest and Norway the lowest number of hours. The figure also highlights clear differences between jobs. “Medical doctors” (ISCO 2221) experience minimal exposure due to low metabolic rates and indoor cooling. Greater variation can be observed in heat stress exposure across regions among “crop and animal producers” (ISCO 6130), who work fully outdoors with a moderate to high metabolic rate, “painters and related workers” (ISCO 7141), who work both indoor and outdoor with a moderate metabolic rate, and “building construction laborers” (ISCO 9313), who work entirely outdoors, perform physically intense tasks, and wear protective helmets that hinder heat dissipation.

### Heat stress exposure across time

**Figure 4:**
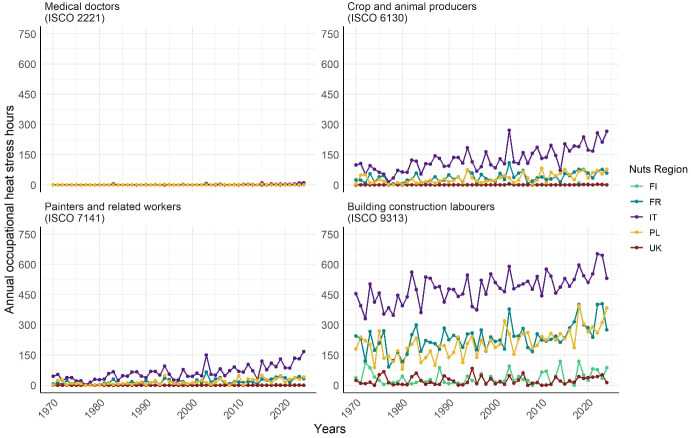
Temporal changes in annual occupational heat stress hours for selected jobs and countries in Europe. Calculations are based on unacclimatized workers with a body surface area of 1.9 m^2^, assuming full-time employment from 09:00–17:00 hours (8 hours/day, 5 days/week) and 30 vacation days (230 workdays/year).

Figure 4 shows that the risk of heat stress in the four selected jobs changes over time, with a general trend of a slight increase. The extent of variation depends on both the job and country, with greater fluctuations observed in those with higher heat stress risk. For instance, among “building construction laborers”, the annual occupational heat stress hours in France ranged from 91 in 1977 to 404 in 2023, while in Poland, the largest variations were observed between 1980 with 80 hours and 2018 with 395 hours. In contrast, in the UK, differences over time were less pronounced, with a maximum difference of 83 hours between 1995 (83 hours) and 1974 (0 hours).

### Sensitivity analysis

**Figure 5:**
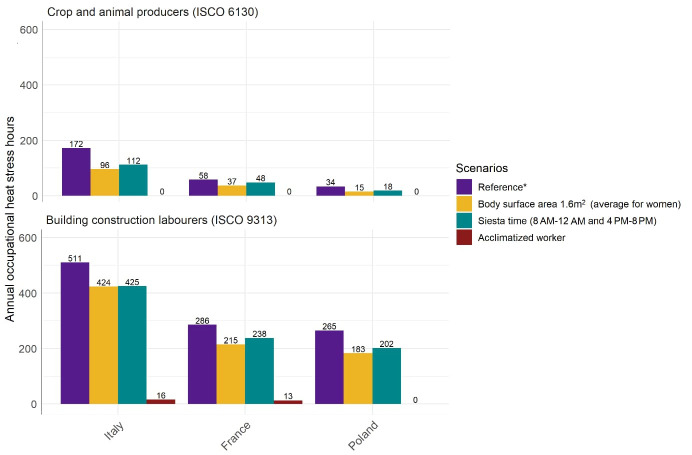
Sensitivity analysis of heat stress exposure under varying input parameters for selected jobs and European countries in 2020. * Reference calculations are based on unacclimatized workers with a body surface area of 1.9 m^2^ (average for men) in 2020, assuming full-time employment from 09:00–17:00 hours (8 hours/day, 5 days/week) and 30 vacation days (230 workdays/year).

Figure 5 summarizes how annual occupational heat stress hours vary under different input parameters. Lowering the body surface area to the average for women (1.6 m^2^ versus 1.9 m^2^ for men) resulted in fewer heat stress hours. For example, among “building construction laborers”, differences of 87 hours in Italy, 71 hours in France, and 82 hours in Poland were observed. Siesta time reduced exposure by avoiding the hottest hours (08:00–12:00 and 16:00–20:00 versus full-time 09:00–17:00 hours). For example, among “building construction laborers”, annual heat stress hours decreased by 86 hours in Italy, 48 hours in France, and 63 hours in Poland. Acclimatization status had the most pronounced effect on reducing heat stress hours, with acclimatized individuals having a WBGT_ref_ that was 2.6°C higher than those who are not acclimatized. Among “building construction laborers”, heat stress exposure dropped by 495 hours in Italy (from 511 to 16), 273 hours in France (from 286 to 16), and 265 hours in Poland (from 265 to 0). For “crop and animal producers”, acclimatization eliminates heat stress exposure entirely, reducing hours from 172 in Italy, 58 in France, and 34 in Poland to 0 across all cases.

## Discussion

We present a heat JEM that enables epidemiological research on occupational heat stress across Europe. The JEM is built on a transparent framework that integrates existing ISO standards on heat stress. It combines historical meteorological data with job-specific factors, derived from pre-existing JEM and input from an international panel of experts in industrial hygiene and exposure assessment. Applying this JEM, we found that occupational heat stress is highest in physically demanding jobs and jobs involving local heat sources, particularly when work clothing is worn. Exposure is most pronounced in Southern Europe, varies across calendar years, and is substantially higher among unacclimatized workers compared to acclimatized workers.

Unlike traditional JEM that typically assign fixed exposure levels by job, the heat JEM developed in this study integrates job-, region-, and year-specific information to account for spatiotemporal variation in heat exposure across Europe. Moreover, its transparent framework allows for further customization by adjusting for work organization, acclimatization status or other modifying factors relevant to the study population. As a result, the JEM is delivered as a flexible R-based algorithm rather than a static matrix, offering greater flexibility for epidemiological research.

A Finnish heat JEM has previously been developed and recently applied to studies conducted in France, Canada, and Spain to investigate heat-related health effects (20, 31). Building on this JEM, a panel of five experienced industrial hygienists adapted it to Spanish working conditions using their expertise in company-based industrial hygiene measurements (21). Like our JEM, both previous JEM defined occupational heat exposure based on ISO 7243 standards, with heat stress occurring when WBGT_eff_ exceeds WBGT_ref_ (22). However, as these earlier JEM did not provide job-specific heat stress hour estimates, direct comparison with our findings is precluded. Moreover, limited methodological transparency, particularly regarding the incorporation of meteorological data and job-specific factors, may restrict the applicability of those models beyond their original contexts.

Our JEM systematically integrates high-resolution meteorological data and detailed occupational characteristics, allowing for adaptability across geographic regions and calendar years. This integration is essential, as our findings underscore that both environmental conditions and job-related factors play a pivotal role in shaping heat stress exposures. For instance, as expected due to the warmer climate, workers in Southern Europe are more frequently exposed to heat stress than those in other parts of the continent. Moreover, workers performing highly physically demanding tasks in warm climates are especially likely to experience heat stress, while those exposed to local heat sources are at a high heat stress risk regardless of their geographic region.

The Finnish heat JEM, applied using 3-year averages from 1995 to 2009, showed minimal temporal variation, leading the authors to average exposure values across the entire study period (31). In contrast, our JEM estimates reveal pronounced temporal variability in occupational heat stress, with significant differences observed not only between countries but also across different jobs within the same country. This variability is likely driven by WBGT_eff_ values frequently fluctuating around the reference threshold (WBGT_ref_), where minor changes in meteorological conditions can shift exposures above the threshold. Consequently, large year-to-year fluctuations in heat stress hours can occur, even when average daily or monthly WBGT values appear relatively stable.

Our transparent framework allows for custom applications in epidemiological studies by incorporating factors such as person-specific body surface area and variations in work hours, thereby enhancing the precision of occupational heat stress assessments. Although detailed individual-level data on these parameters are often unavailable in cohorts, many can be approximated using region-specific information. For instance, we have demonstrated that acclimatized workers, those who have adapted to heat, have a considerably lower risk of heat stress, due to a WBGT_ref_ that is 2.6°C higher than that of non-acclimatized individuals. Given that acclimatization typically develops after several consecutive days of heat exposure, it is reasonable to assume that workers in Southern European countries are generally acclimatized during the hottest months of the year (32, 33). Our findings also highlight the importance of work timing in determining occupational heat stress, which can vary by region. For instance, in Nordic countries, long summer holidays typically overlap with the warmest period of the year, and midday siestas in certain Southern European regions shift work away from the hottest hours. Incorporating such region-specific practices can improve the accuracy of heat stress estimates in the JEM.

In the current version of the heat JEM, sex-specific differences are accounted for only through variation in body surface area, which is used to estimate metabolic rate. As men typically have larger body surface area than women, they are assigned higher metabolic rates and thus a higher estimated risk of heat stress. Nevertheless, other physiological sex differences also influence heat response, including generally higher sweat rates among men that enhance heat dissipation and hormonal differences among women that affect thermoregulation (34). Differences in body composition and aerobic capacity may also lead to greater strain among women when performing the same physical tasks under similar heat exposure conditions (34, 35). Moreover, individual characteristics like age, pregnancy, medication use, and disease status can affect heat tolerance (36–38). Although these influences are well recognized, there is no clear consensus on how to incorporate them into the heat exposure assessment framework.

The strengths of our study include its transparent and adaptable framework, alignment with internationally recognized ISO standards, and integration of job-specific factors including clothing, physical activity level, time spent indoors versus outdoors, and local heat or cooling sources. These factors were derived from existing datasets and refined by a panel of five experts with complementary expertise in occupational hygiene, exposure assessment, and thermal physiology. This approach ensures consistency, relevance and practical applicability across jobs. The JEM is further strengthened by the use of high-resolution meteorological data from ECMWF ERA5-Land, covering 1970–2024, with hourly estimates of key climate variables and the ability to incorporate future updates.

While our JEM incorporates numerous advancements, its development required several assumptions that may influence exposure estimates. First, as time spent in shaded environments was not considered, individual exposure is likely overestimated. Second, to estimate indoor WBGT, we assumed zero irradiance and a wind speed of 0.4 m/s, which aligns with typical indoor conditions but may not fully capture building-specific factors such as strong air circulation or radiant heat sources (22). Third, calculations relied on average physical activity levels to determine metabolic rate per job, without accounting for short-term fluctuations (23). Given the right-skewed distribution of physical activity, this approach likely overestimates WBGT_ref_, as short periods of high physical activity can disproportionately inflate average values. Fourth, we lack information on the specific hours during which workers are exposed to local heat sources and, for firefighters, the hours they wear their work clothing throughout the day. Therefore, we currently assume these exposures are evenly distributed across the workday. Fifth, our expert panel included ratings from Northern, Western, and Southern European countries, but lacked representation from Eastern Europe. Given that only minor differences were identified between Southern and Northern/Western Europe in local heat and cooling source ratings, substantial variation in Eastern Europe appears unlikely, although it cannot be ruled out. Fifth, we did not account for individual-level job control, which can allow workers to manage heat exposure (eg, by slowing down, taking breaks, or seeking cooler areas). This may lead to overestimation of exposure in settings with high worker autonomy. Lastly, the JEM has not yet been validated. However, within the ongoing EU-INTERCAMBIO project (39, 40), personal temperature and humidity measurements, stationary WBGT measurements, physical activity data, and questionnaire and contextual data (eg, clothing, cooling sources) are currently being collected across various priority occupational settings in Europe to support future validation of model inputs and exposure estimates.

### Concluding remarks

The heat JEM provides a structured and comprehensive framework for assessing occupational heat stress exposure across jobs, regions, and years in epidemiological research. We found that exposure is highest in physically demanding jobs and jobs involving local heat sources, particularly when work clothing is mandatory. Exposure is most pronounced in Southern Europe, varies across calendar years, and is substantially higher among unacclimatized workers. The JEM’s transparent framework allows for incorporation of new or more detailed cohort-specific data when available, and provides a basis for extension to additional years and geographic regions beyond Europe.

## Supplementary material

Supplementary material
