# Supplementary material for: Exposure to heat at work: development of a quantitative European job exposure matrix (heat JEM)
Source: Scand J Work Environ Health. 2025 Dec 27;52(1):7–18. doi: 10.5271/sjweh.4243 (PMC12767608; doi:10.5271/sjweh.4243)
Supplement: Supplementary material [file SJWEH-52-7-S001.pdf]

Exposure to heat at work: development of a quantitative European job exposure matrix (heat JEM)[1]

by Tosca OE de Crom, PhD,[2] Bernice Scholten, PhD, Eugenio Traini, PhD, Koen van der Sanden, MSc, Boris Kingma, PhD, Floris Pekel, MSc, Manosij Ghosh, PhD, Hilde Notø, PhD, Michelle C Turner, PhD, Miguel Angel Alba Hidalgo, PhD, Lisa Klous, PhD, Maria Albin, PhD, Henrik A Kolstad, PhD, Jenny Selander, PhD, Calvin Ge, PhD, Anjoeka Pronk, PhD

1. Supplementary Material
2. Correspondence to: Tosca OE de Crom, Department of Risk Analysis for Prevention, Netherlands Organisation for Applied Scientific Research TNO, Princetonlaan 6, 3584 CB Utrecht, The Netherlands. [E-mail: tosca.decrom@tno.nl]

Supplementary Methods

Step 1. Outdoor and indoor WBGT

Outdoor and indoor WBGT values were calculated separately based on the fifth-generation reanalysis land (ERA5-Land) meteorological data derived from the European Centre for Medium Range Weather Forecasts (ECMWF), which include hourly data throughout Europe at a resolution of 0.1 by 0.1 latitude-longitude (approximately 9 by 9 km) between 1970 and 2024 (1). Meteorological data used for these calculations include air temperature (dry-bulb ambient temperature), relative humidity (wet-bulb temperature), wind speed (wet-bulb temperature and globe temperature), and radiant heat sources (wet-bulb temperature and globe temperature). The complete list of ERA5-Land variables used in the WBGT calculation can be found in Supplementary Table 1. These variables were downloaded between September 2024 and April 2025.

Natural wet-bulb temperature (tnw) and globe temperature (tg) were estimated using the Liljegren method based on meteorological data (2). These estimates, along with dry-bulb ambient temperature (ta), were subsequently used to calculate outdoor WBGT using the standard ISO 7243 formula:

$$\text{Outdoor WBGT} = 0.7 * t_{nw} + 0.2 * t_g + 0.1 * t_a$$

Indoor WBGT was calculated by assuming that the radiant temperature equals the dry bulb temperature, wind speed is 0.4 meters per second, and irradiance is zero for the calculation of the natural wet-bulb temperature, which simplifies the formula to:

$$\text{Indoor WBGT} = 0.7 * t_{nw} + 0.3 * t_a$$

Hourly WBGT values were calculated at the Nomenclature of Territorial Units for Statistics (NUTS) 3 level. The ERA5 dataset provides hourly data in Coordinated Universal Time (UTC), also known as Greenwich Mean Time (GMT), which was used in the model. Finally, WBGT values were adjusted to the appropriate local time zones based on the NUTS classification.

Data processing and analysis of WBGT values were conducted using Python statistical software.

Step 2. Outdoor and indoor $WBGT_{eff}$

The outdoor and indoor WBGT values obtained in step 1 were modified by job-specific clothing adjustment values (CAVs) to obtain the $WBGT_{eff}$. The CAVs were assigned to each ISCO-88(COM) job code by the expert panel through categorizing jobs based on mandatory work clothing and head covering requirements. The corresponding CAVs were then applied according to ISO 9920 (3). using the following formula:

$WBGT_{eff} = WBGT + CAV$

Supplementary Table 2 provides an overview of the clothing categories and head covering classifications, and their corresponding CAVs. Three clothing categories were distinguished, i.e. low (normal work clothes, cloth coveralls), medium (non-woven or double layer coveralls or vapour barrier aprons) and high (vapor barrier coveralls or firefighting turnout gear). To each category, a CAV was assigned: 0 °C-WBGT for low, +3 °C-WBGT for medium, and +11 °C-WBGT for high. For the high clothing category, an additional assessment was conducted by the experts to determine how frequently workers wear that specific outfit. This consideration was deemed relevant exclusively for firefighters, who were classified to wear their full protective outfit for a randomly distributed average of 6 hours per 40-hour working week during activities spent outdoors. In addition, the mandatory use of a protective helmet or a hood was assessed by the expert panel, for which an additional CAV value of +1 °C-WBGT was assigned. Caps that could protect against sunshine, hairnets, or chef's hats were not considered relevant for heat assessment. These categories and related CAVs were based on ISO 7243 (4) and the Occupational Safety and Health Administration (OSHA) clothing adjustment factors which were adopted from the American Conference of Governmental Industrial Hygienists (ACGIH) “2017 Threshold Limit Values (TLVs) and Biological Exposure Indices (BEIs)” (5).

Step 3. $WBGT_{ref}$

$WBGT_{ref}$ were calculated based on the metabolic rate specific to each job, estimated according to ISO 8996 (6), and the acclimatization status. The calculations were performed using the following formula:

Unacclimatized workers: $WBGT_{ref} = 59.9 – 14.1 * log_{10}(metabolic\ rate)$

Acclimatized workers: $WBGT_{ref} = 59.9 – 11.5 * log_{10}(metabolic\ rate)$

Metabolic rate was determined using physical activity, expressed in metabolic equivalent of task (MET, 1 MET ~ 58.2 Watts per m^{-2}) (6). Quantitative job-specific MET values were derived from a previously developed JEM as described in detail elsewhere (7). Briefly, for this JEM, MET values were assigned to job-specific tasks compiled for each ISCO-08 code. The MET values corresponding to these tasks were averaged to obtain an average hourly MET value per ISCO-08 code, which were subsequently converted to ISCO-88(COM) codes using a crosswalk developed by an expert in occupational epidemiology (Calvin Ge).

For the heat JEM, metabolic rates in Watts were calculated based on these MET values using the formula:

Metabolic rate = MET value × body surface area × 58.2,

where body surface area in m^2 was estimated using the Du Bois formula (8):

$Body\ surface\ area = 0.007184 \times weight\ (kg)^{0.425} \times height\ (cm)^{0.725}$.

If information on weight and height is unavailable, typical sex-specific body surface area values can be used—approximately 1.9 m^2 for men and 1.6 m^2 for women (9)—though these may vary slightly depending on population characteristics (10, 11). This approach implicitly assumes that women are less vulnerable to heat stress due to their lower average body surface area. We did not apply any further sex-specific adjustments to the $WBGT_{ref}$, as existing evidence on sex-based differences in vulnerability to heat stress is inconsistent and no standardized adjustment values have been established (12).

Step 4. Heat stress hours classification ($WBGT_{eff} > WBGT_{ref}$)

After obtaining $WBGT_{eff}$ for each hour and $WBGT_{ref}$ as a standard across all hours, heat stress was determined for each outdoor and indoor hour by evaluating whether $WBGT_{eff}$ exceeds $WBGT_{ref}$ (4).

Step 5. Indoor heat stress hours adjusted for local heat and cooling sources

For indoor work, further adjustments were made for the presence of local heat and cooling sources. During the hours workers were exposed to a local heat source while performing indoor tasks, the indoor $WBGT_{eff}$ were overruled, and these exposure hours were classified as heat stress. Conversely, in the presence of local cooling, it was assumed that the indoor environment is sufficiently cooled below $WBGT_{ref}$, resulting in no exposure to heat stress. Under this assumption, local cooling overrules the effect of meteorological conditions and local heat sources.

The presence of a local heat source as well as the worker's likelihood and duration of exposure were assessed using a two-step approach. First, a literature search was conducted to assess publications on occupational heat sources and occupational heat exposure in 2023. From this review, 64 articles (including duplicates) were examined to identify heat sources. This search was subsequently expanded with reports from OSHA and EU-OSHA, non-scientific documents and websites that provide additional information on potential heat sources per industry or occupation. An overview of identified heat sources is provided in Supplementary Table 3. Based on this literature overview, an initial assessment of the potential presence of each heat source was made for each ISCO-88(COM) job code by the researcher who reviewed the literature (FP). Second, the five experts of the expert panel reviewed this initial assessment independently by confirming or rejecting the assessment of the proposed heat source in that specific job. Experts also rated the likelihood of exposure to the heat source and the exposure duration. The likelihood of exposure was categorized into four levels by the percentage of workers with that job affected by a heat source: approximately 10%, 25%, 75%, or 90%. The area that is affected by the local heat sources was defined as within a radius of 5 meter of a non-industrial size heat source, and 10 meters in case of an industrial size source. The exposure duration within the likelihood of the heat source was also categorized into four levels based on average number of working hours per day: <1, 1-2, 3-4, or >4 hours out of an 8-hour work-shift. The total number of exposure hours was determined by taking the midpoint of each frequency category (0.5 hours, 1.5 hours, 3.5 hours, and 6 hours within an 8-hour work shift) and multiplying it by the likelihood of exposure. It was assumed that exposure to local heat sources is evenly distributed across the workday. For the assessment of the presence and frequency of local heat sources, no distinction was made between European countries, as experts concluded that exposure did not

differ across countries. However, for the categorisation of exposure duration, a distinction was made between Southern European countries and other European countries, with Southern European countries defined according to the United Nations geoscheme. Specifically, longer exposure durations were assigned to drivers and heavy machinery operators in Southern European countries.

The worker's likelihood of exposure to active cooling was assessed for all ISCO-88(COM) job codes by the expert panel in four categories: approximately 10%, 25%, 75%, or 90%. Active cooling is defined as the presence of a type of a fan, air conditioning, or HVAC (Heating ventilation and air conditioning) system in the location or area where work is performed. No distinction was made regarding the type of local cooling source or number of hours workers were exposed to active cooling, as it was assumed to be constantly activated when relevant. All workers classified within the 75% and 90% likelihood categories were assumed to be in an indoor environment sufficiently cooled below $WBGT_{ref}$, resulting in no exposure to heat stress. Worker in the lower likelihood categories were assumed to have no indoor cooling.

<u>Step 6. Heat stress hours adjusted for outdoor and indoor work</u>

To account for the ratio of time workers spent indoors versus outdoors, the total annual working hours in which $WBGT_{eff}$ exceeded $WBGT_{ref}$ were first calculated separately for indoor and outdoor conditions (step 1-5 and 1-4, respectively). These values were then weighted by the proportion of time spent indoors and outdoors. The estimation of working hours spent indoors versus outdoors was based on an expert assessment from a previously published JEM for UV exposure (13). In this assessment, six experts assessed the duration of outdoor work per ISCO-88(COM) job title, assuming an 8-hour workday, using a five-point scale, i.e., 0 = 0 hours, 1 = 1–2 hours, 2 = 3–4 hours, 3 = ≥5 hours. After initial ratings, experts discussed disagreements and subsequently independently re-rated each job. These final ratings were averaged and rounded. For the Heat JEM, an additional single expert assessment (CG) was conducted to further classify jobs initially rated as >4 hours outdoors into two categories: 6 hours or a full workday. The average values for each category were used to estimate the percentage of working hours spent outdoors. The final classifications were: 0 hours (0%, i.e. fully indoor), 1.5 hours (18.75%), 3.5 hours (43.75%), 6 hours (75%), and a full workday (100%).

<u>Step 7. Total heat stress hours adjusted for work organization factors</u>

In this final step, the total annual hours in which $WBGT_{eff}$ exceeded $WBGT_{ref}$ were first summed to obtain the total annual exposed hours. This number was then adjusted for work organization conditions, including daily and weekly working hours and vacation days, can be tailored according to cohort-specific practices and norms. Daily working hours refer to the specific time range during which work is performed, while weekly working hours account for the total number of hours worked per week. Vacation days include both the total number of days off and, when specified, the timing.

REFERENCES

European Centre for Medium-Range Weather Forecasts (ECMWF). ERA5-Land hourly data from 1950 to present [Internet]. 2025 [cited 2025 May 14]. Available from: https://cds.climate.copernicus.eu/datasets/reanalysis-era5-land?tab=overview

Liljegren JC, Carhart RA, Lawday P, Tschopp S, Sharp R. Modeling the wet bulb globe temperature using standard meteorological measurements. J Occup Environ Hyg. 2008;5(10):645–55.

International Organization for Standardization (ISO). ISO 9920:2007. Ergonomics of the thermal environment — Estimation of thermal insulation and water vapour resistance of a clothing ensemble. Geneva: ISO; 2007.

International Organization for Standardization (ISO). ISO 7243:2017. Ergonomics of the thermal environment — Assessment of heat stress using the WBGT (wet bulb globe temperature) index. Geneva: ISO; 2017.

Occupational Safety and Health Administration (OSHA). OSHA Technical Manual (OTM), Section III: Chapter 4 [Internet]. 2017 [cited 2024 Jul 31]. Available from: https://www.osha.gov/otm/section-3-health-hazards/chapter-4#clothing

International Organization for Standardization (ISO). ISO 8996:2021. Ergonomics of the thermal environment — Determination of metabolic rate. Geneva: ISO; 2021.

Deyaert J, Harms T, Weenas D, Gershuny J, Glorieux I. Attaching metabolic expenditures to standard occupational classification systems: perspectives from time-use research. BMC Public Health. 2017;17(1):620.

Du Bois D, Du Bois EF. A formula to estimate the approximate surface area if height and weight be known. Arch Intern Med. 1916;17(6):863–71.

RxList. Medical definition of body surface area [Internet]. n.d. [cited 2025 May 14]. Available from: https://www.rxlist.com/body_surface_area/definition.htm

Danesi V, Andalo A, Cavallucci M, Balzi W, Gentili N, Altini M, et al. Body weight and body surface area of adult patients with selected cancers: an Italian multicenter study. PLoS One. 2024;19(12):e0314452.

Tikuisis P, Meunier P, Jubenville CE. Human body surface area: measurement and prediction using three dimensional body scans. Eur J Appl Physiol. 2001;85(3–4):264–71.

Alele F, Malau-Aduli B, Malau-Aduli A, Crowe M. Systematic review of gender differences in the epidemiology and risk factors of exertional heat illness and heat tolerance in the armed forces. BMJ Open. 2020;10(4):e031825.

Wurtz ET, Pugdahl K, Fenger-Gron M, Berglind IA, Cherrie MPC, Dahlman-Hoglund A, et al. A quantitative solar ultraviolet radiation job-exposure matrix for Europe. Ann Work Expo Health. 2025.

Supplementary Table S1. Overview of ERA5-Land variables used in WBGT calculation with descriptions.

ERA5-Land variables	Description
2m dewpoint temperature	Temperature to which the air, at 2 meters above the surface of the Earth, would have to be cooled for saturation to occur. It is a measure of the humidity of the air.
2m temperature	Temperature of air at 2m above the surface of land, sea or in-land waters.
Surface net solar radiation	Amount of solar radiation (also known as shortwave radiation) reaching the surface of the Earth (both direct and diffuse) minus the amount reflected by the Earth's surface (which is governed by the albedo)
Surface solar radiation downwards	Amount of solar radiation (also known as shortwave radiation) reaching the surface of the Earth. This variable comprises both direct and diffuse solar radiation).
10m u-component of wind	Eastward component of the 10m wind
10m v-component of wind	Northward component of the 10m wind
Surface pressure	Pressure (force per unit area) of the atmosphere on the surface of land, sea and in-land water. It is a measure of the weight of all the air in a column vertically above the area of the Earth's surface represented at a fixed point

ERA5-Land: Fifth-generation ECMWF atmospheric reanalysis dataset for land applications.

Supplementary Table S2. Overview of clothing categories and assigned CAV values.

Clothing category	Description	CAV values (°C-WBGT)
Low	Work clothes made from a woven fabric, considered the reference ensemble.<br>Cloth coveralls made from woven fabric, including treated cotton.<br>Non-woven SMS (Spunbond-Meltblown-Spunbond) coveralls (single layer) from a non-proprietary fabric made from polypropylene.	0
Moderate	Non-woven polyolefin coveralls (single layer) from proprietary polyethylene fabric, such as micro-porous materials (e.g., Tyvek™).<br>Vapor-barrier apron with long sleeves and long length over cloth coveralls, designed as a wrap-around apron to protect the front and sides from chemical spills.<br>Double layer of woven clothing, typically consists of coveralls worn over standard work clothes.	+ 3
High	Limited-use vapor-barrier coveralls, i.e. full-body protective garments designed for extreme environments. Examples include encapsulating suits, whole-body chemical protective suits, firefighter turnout gear.	+ 11
Protective helmet or hood	Mandatory use of a protective helmet or hood which generally adds to the heat burden. This does not include, for instance, a cap for sun protection, a hairnet, or a chef's hat.	+ 1

The clothing categories and corresponding clothing adjustment values (CAVs) were based on ISO 7243 and the Occupational Safety and Health Administration (OSHA) clothing adjustment factors, which were adopted from the American Conference of Governmental Industrial Hygienists (ACGIH) "2017 Threshold Limit Values (TLVs) and Biological Exposure Indices (BEIs)".

Supplementary Table S3. Overview of identified heat sources.

Source
Industrial Oven
Industrial Furnace
Industrial Smelter
Industrial Kiln
Other industrial heat source
Retail/ Workshop-size Oven
Retail/ Workshop-size Furnace
Retail/ Workshop-size Kiln
Retail/ Workshop-size Other heat source
Heated material
Boiler
Greenhouse effect
Welding equipment
Kitchen equipment
Engine - Engine room
Drying / Cleaning equipment
Geothermal heating
Heated environment (e.g. Roof / Asphalt)
Vehicle cabin
Incinerator
Canning

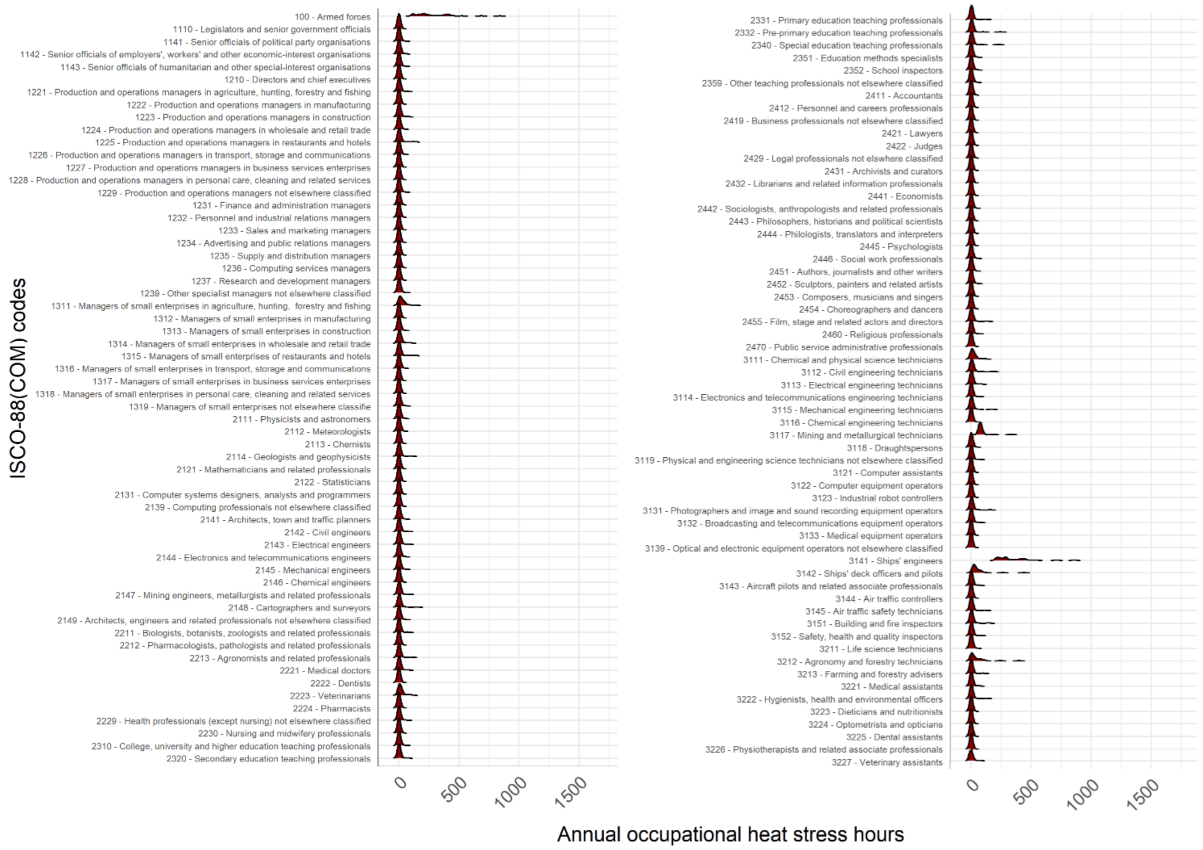

Supplementary Figure S1a. Ridge plot depicting variation in annual occupational heat stress hours across all jobs and European countries. *Each ridge represents the variation in annual occupational heat stress hours across* 37 European countries *for the corresponding ISCO-88(COM) code listed on the y-axis, covering codes from 100 to 4223. Higher peaks indicate that most workers within an job experience similar heat stress hours across regions, whereas wider distributions reflect greater variation in heat stress hours across regions within that job. Calculations are based on unacclimatized workers with a body surface area of 1.9 m^2 in 2020, assuming full-time employment from 9 AM to 5 PM (8 hours/day, 5 days/week) and 30 vacation days (230 workdays/year).*

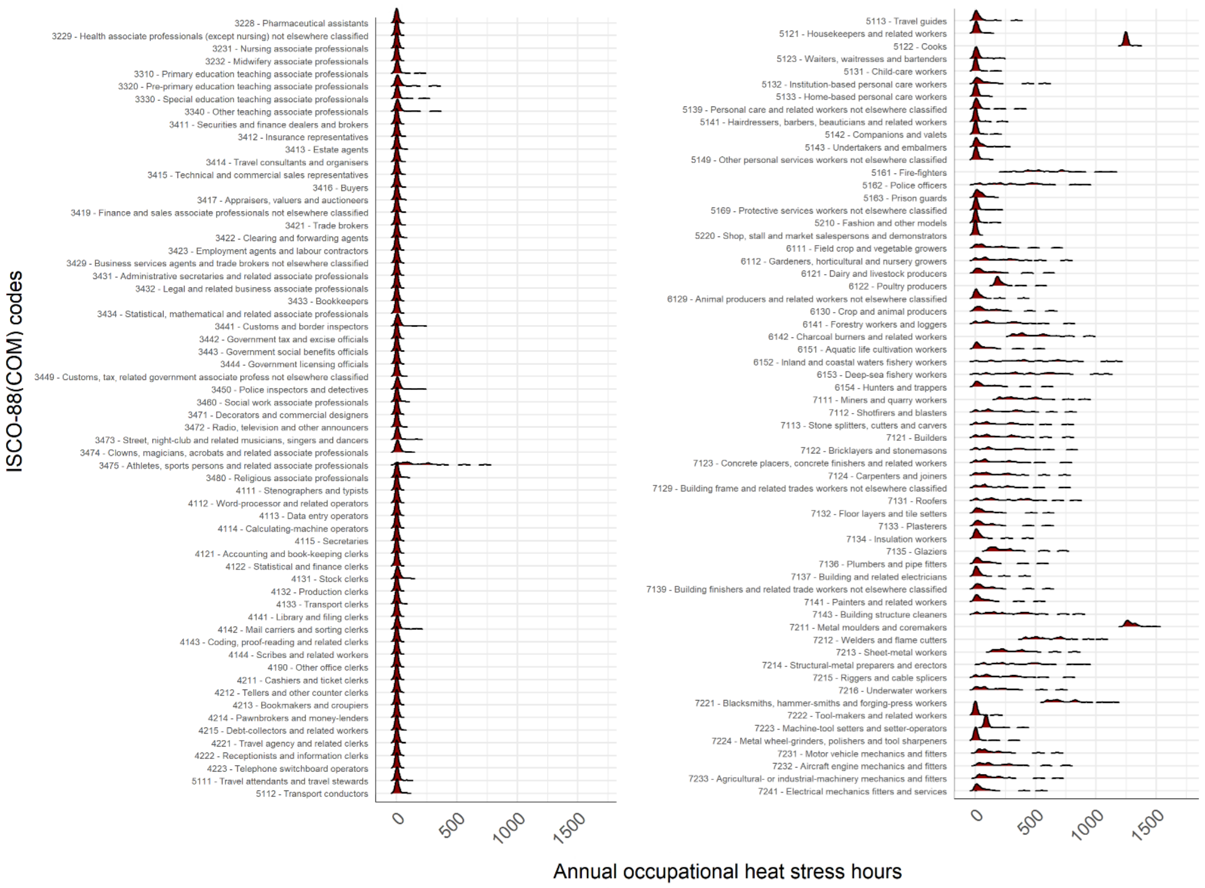

Supplementary Figure S1b. Ridge plot depicting variation in annual occupational heat stress hours across all jobs and European countries. *Each ridge represents the variation in annual occupational heat stress hours across 37 European countries for the corresponding ISCO-88(COM) code listed on the y-axis, covering codes from 5111 to 9330. Higher peaks indicate that most workers within an job experience similar heat stress hours across regions, whereas wider distributions reflect greater variation in heat stress hours across regions within that job. Calculations are based on unacclimatized workers with a body surface area of 1.9 m^2 in 2020, assuming full-time employment from 9 AM to 5 PM (8 hours/day, 5 days/week) and 30 vacation days (230 workdays/year).*

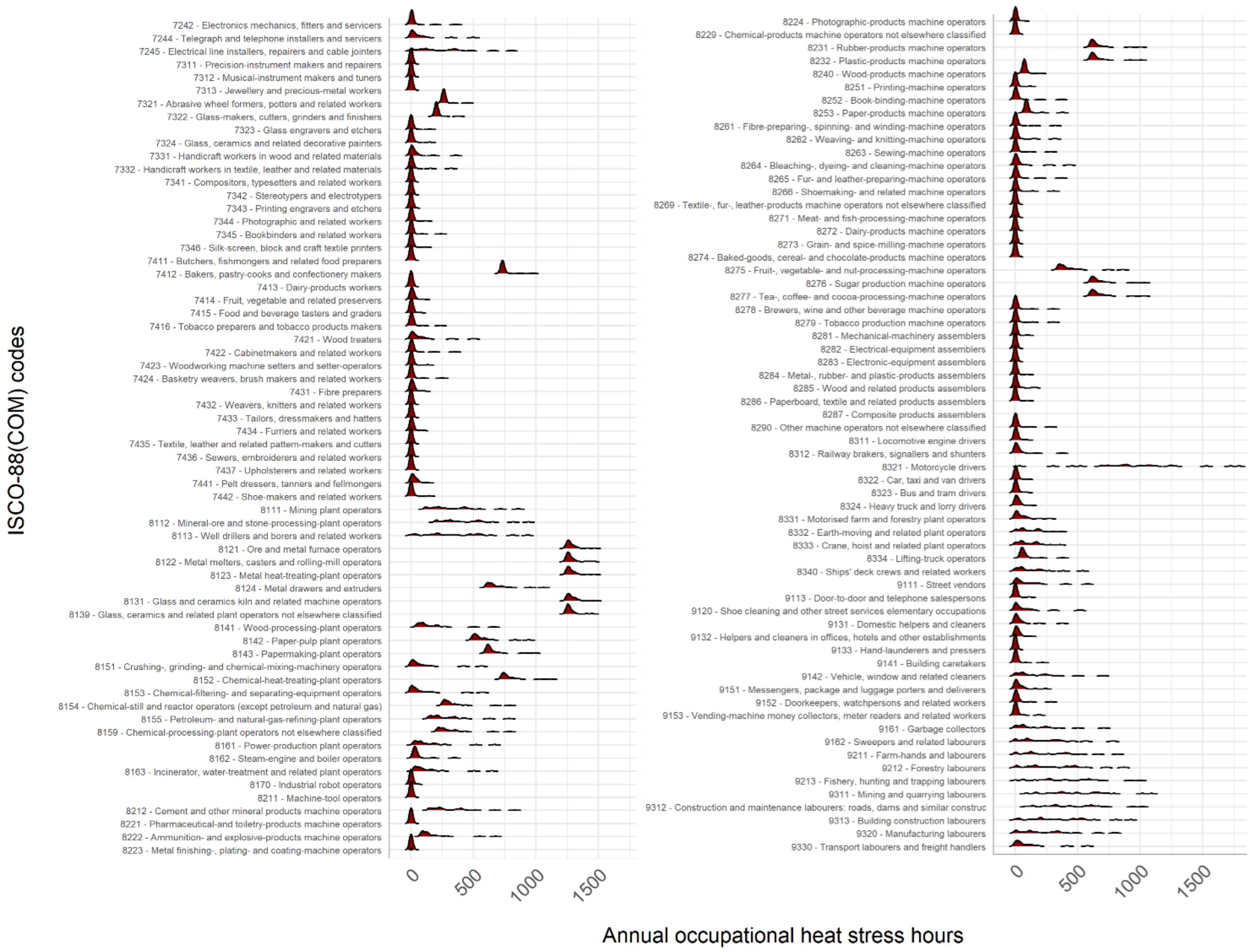

Supplementary Figure S1c. Ridge plot depicting variation in annual occupational heat stress hours across all jobs and European countries. *Each ridge represents the variation in annual occupational heat stress hours across 37 European countries for the corresponding ISCO-88(COM) code listed on the y-axis, covering codes from 5111 to 9330. Higher peaks indicate that most workers within an job experience similar heat stress hours across regions, whereas wider distributions reflect greater variation in heat stress hours across regions within that job. Calculations are based on unacclimatized workers with a body surface area of 1.9 m^2 in 2020, assuming full-time employment from 9 AM to 5 PM (8 hours/day, 5 days/week) and 30 vacation days (230 workdays/year).*

Supplementary Table S4a. Step-by-step example of heat stress hours calculation using the heat JEM (medical doctors, ISCO 2221).

<table>
<tr><th>Steps</th><th>Description</th></tr>
<tr><td>Step 1. Outdoor and indoor WBGT</td><td>As an example, the table below presents indoor and outdoor WBGT values (°C) for standard work hours (9:00 AM to 5:00 PM) on 1 August 2020 in France (NUTS 0 level).
<table>
<tr><th>Time</th><th>Outdoor WBGT (°C)</th><th>Indoor WBGT (°C)</th></tr>
<tr><td>9:00 – 10:00 AM</td><td>21.20</td><td>20.01</td></tr>
<tr><td>10:00 – 11:00 AM</td><td>22.52</td><td>20.77</td></tr>
<tr><td>11:00 – 12:00 PM</td><td>23.59</td><td>21.54</td></tr>
<tr><td>12:00 – 1:00 PM</td><td>24.50</td><td>22.29</td></tr>
<tr><td>1:00 – 2:00 PM</td><td>25.04</td><td>22.92</td></tr>
<tr><td>2:00 – 3:00 PM</td><td>25.29</td><td>23.34</td></tr>
<tr><td>3:00 – 4:00 PM</td><td>25.30</td><td>23.55</td></tr>
<tr><td>4:00 – 5:00 PM</td><td>25.07</td><td>23.51</td></tr>
</table>
</td></tr>
<tr><td>Step 2. Outdoor and indoor $WBGT_{eff}$</td><td>For “medical doctors”, the expert panel assumed no mandatory work clothing or head covering is worn, meaning that WBGT values were taken as $WBGT_{eff}$ values.
<table>
<tr><th>Time</th><th>Outdoor $WBGT_{eff}$ (°C)</th><th>Indoor $WBGT_{eff}$ (°C)</th></tr>
<tr><td>9:00 – 10:00 AM</td><td>21.20</td><td>20.01</td></tr>
<tr><td>10:00 – 11:00 AM</td><td>22.52</td><td>20.77</td></tr>
<tr><td>11:00 – 12:00 PM</td><td>23.59</td><td>21.54</td></tr>
<tr><td>12:00 – 1:00 PM</td><td>24.50</td><td>22.29</td></tr>
<tr><td>1:00 – 2:00 PM</td><td>25.04</td><td>22.92</td></tr>
<tr><td>2:00 – 3:00 PM</td><td>25.29</td><td>23.34</td></tr>
<tr><td>3:00 – 4:00 PM</td><td>25.30</td><td>23.55</td></tr>
<tr><td>4:00 – 5:00 PM</td><td>25.07</td><td>23.51</td></tr>
</table>
</td></tr>
<tr><td>Step 3. $WBGT_{ref}$</td><td>The activity level of “medical doctors”, corresponds to 1.85 metabolic equivalents (METs). The metabolic rate was calculated as:
Metabolic rate = MET * body surface area * 58.2

Using a body surface area of 1.9 m^2, this yields:
1.85 * 1.9 * 58.2 = 204.6 Watts

The $WBGT_{ref}$ value is than calculated as:
$WBGT_{ref} = 59.9 – 14.1 \times \log_{10}(\text{metabolic rate})$
Which results in:
$WBGT_{ref} = 59.9 – 14.1 \times \log_{10}(204.6) = 27.32$°C</td></tr>
<tr><td>Step 4. Heat stress hours classification ($WBGT_{eff} > WBGT_{ref}$)</td><td>Each hour is classified as a heat stress hour if $WBGT_{eff} > WBGT_{ref}$:
<table>
<tr><th>Time</th><th>Outdoor $WBGT_{eff}$ (°C)</th><th>WBGTref (°C)</th><th>Outdoor $WBGT_{eff} > WBGT_{ref}$</th></tr>
<tr><td>9:00 – 10:00 AM</td><td>21.20</td><td>27.32</td><td>No</td></tr>
<tr><td>10:00 – 11:00 AM</td><td>22.52</td><td>27.32</td><td>No</td></tr>
<tr><td>11:00 – 12:00 PM</td><td>23.59</td><td>27.32</td><td>No</td></tr>
<tr><td>12:00 – 1:00 PM</td><td>24.50</td><td>27.32</td><td>No</td></tr>
<tr><td>1:00 – 2:00 PM</td><td>25.04</td><td>27.32</td><td>No</td></tr>
<tr><td>2:00 – 3:00 PM</td><td>25.29</td><td>27.32</td><td>No</td></tr>
</table>
</td></tr>
</table>

	3:00 – 4:00 PM	25.30	27.32	No<br>4:00 – 5:00 PM	25.07	27.32	No

Time	Indoor $WBGT_{eff}$ (°C)	WBGTref (°C)	Indoor $WBGT_{eff}$ > $WBGT_{ref}$
9:00 – 10:00 AM	20.01	27.32	No
10:00 – 11:00 AM	20.77	27.32	No
11:00 – 12:00 PM	21.54	27.32	No
12:00 – 1:00 PM	22.29	27.32	No
1:00 – 2:00 PM	22.92	27.32	No
2:00 – 3:00 PM	23.34	27.32	No
3:00 – 4:00 PM	23.55	27.32	No
4:00 – 5:00 PM	23.51	27.32	No

Step 5. Indoor heat stress hours adjusted for local heat and cooling sources	The expert panel assumed that “medical doctors” are not exposed to local heat sources but are exposed to local cooling sources. Therefore, if indoor $WBGT_{eff}$ values exceeded $WBGT_{ref}$, this was not considered to represent actual heat stress exposure.
Steps 1 to 5 are repeated for each individual work hour to estimate total annual occupational heat stress exposure.	
Step 6. Heat stress hours adjusted for outdoor and indoor work	Across the full year (including weekends), 23 hours exceeded the $WBGT_{ref}$ threshold for outdoor work, and 0 hours for indoor work. “Medical doctors” are classified as working 18.75% of their time outdoor and 81.25% of their time indoor. Therefore, the total number of heat stress hours is calculated as:<br><br>(23 * 0.1875) + (0 * 0. 8125) = 4 hours
Step 7. Total heat stress hours adjusted for work organization factors	To reflect a typical work schedule of five working days per week and 30 vacation days per year (230 work hours per year), the total number of heat stress hours was adjusted proportionally:<br><br>4 * 230 / 365 = 3 hours

Supplementary Table S4b. Step-by-step example of heat stress hours calculation using the heat JEM (crop and animal producers, ISCO 6130).

<table>
<tr><th>Steps</th><th>Description</th></tr>
<tr><td>Step 1. Outdoor and indoor WBGT</td><td>As an example, the table below presents indoor and outdoor WBGT values (°C) for standard work hours (9:00 AM to 5:00 PM) on 1 August 2020 in France (NUTS 0 level).
<table>
<tr><th>Time</th><th>Outdoor WBGT (°C)</th><th>Indoor WBGT (°C)</th></tr>
<tr><td>9:00 – 10:00 AM</td><td>21.20</td><td>20.01</td></tr>
<tr><td>10:00 – 11:00 AM</td><td>22.52</td><td>20.77</td></tr>
<tr><td>11:00 – 12:00 PM</td><td>23.59</td><td>21.54</td></tr>
<tr><td>12:00 – 1:00 PM</td><td>24.50</td><td>22.29</td></tr>
<tr><td>1:00 – 2:00 PM</td><td>25.04</td><td>22.92</td></tr>
<tr><td>2:00 – 3:00 PM</td><td>25.29</td><td>23.34</td></tr>
<tr><td>3:00 – 4:00 PM</td><td>25.30</td><td>23.55</td></tr>
<tr><td>4:00 – 5:00 PM</td><td>25.07</td><td>23.51</td></tr>
</table></td></tr>
<tr><td>Step 2. Outdoor and indoor $WBGT_{eff}$</td><td>For “crop and animal producers” the expert panel assumed no mandatory work clothing or head covering is worn, meaning that WBGT values were taken as $WBGT_{eff}$ values.
<table>
<tr><th>Time</th><th>Outdoor $WBGT_{eff}$ (°C)</th><th>Indoor $WBGT_{eff}$ (°C)</th></tr>
<tr><td>9:00 – 10:00 AM</td><td>21.20</td><td>20.01</td></tr>
<tr><td>10:00 – 11:00 AM</td><td>22.52</td><td>20.77</td></tr>
<tr><td>11:00 – 12:00 PM</td><td>23.59</td><td>21.54</td></tr>
<tr><td>12:00 – 1:00 PM</td><td>24.50</td><td>22.29</td></tr>
<tr><td>1:00 – 2:00 PM</td><td>25.04</td><td>22.92</td></tr>
<tr><td>2:00 – 3:00 PM</td><td>25.29</td><td>23.34</td></tr>
<tr><td>3:00 – 4:00 PM</td><td>25.30</td><td>23.55</td></tr>
<tr><td>4:00 – 5:00 PM</td><td>25.07</td><td>23.51</td></tr>
</table></td></tr>
<tr><td>Step 3. $WBGT_{ref}$</td><td>The activity level of “building construction labourers” corresponds to 3.15 metabolic equivalents (METs). The metabolic rate was calculated as:
Metabolic rate = MET * body surface area * 58.2

Using a body surface area of 1.9 m^2, this yields:
3.15 * 1.9 * 58.2 = 348.3 Watts

The $WBGT_{ref}$ value is than calculated as:
$WBGT_{ref} = 59.9 – 14.1 \times \log_{10}(\text{metabolic rate})$
Which results in:
$WBGT_{ref} = 59.9 – 14.1 \times \log_{10}(348.3) = 24.06$ °C</td></tr>
<tr><td>Step 4. Heat stress hours classification ($WBGT_{eff} > WBGT_{ref}$)</td><td>Each hour is classified as a heat stress hour if $WBGT_{eff} > WBGT_{ref}$:
<table>
<tr><th>Time</th><th>Outdoor $WBGT_{eff}$ (°C)</th><th>WBGTref (°C)</th><th>Outdoor $WBGT_{eff} > WBGT_{ref}$</th></tr>
<tr><td>9:00 – 10:00 AM</td><td>22.20</td><td>24.06</td><td>No</td></tr>
<tr><td>10:00 – 11:00 AM</td><td>23.52</td><td>24.06</td><td>No</td></tr>
<tr><td>11:00 – 12:00 PM</td><td>24.59</td><td>24.06</td><td>Yes</td></tr>
<tr><td>12:00 – 1:00 PM</td><td>25.50</td><td>24.06</td><td>Yes</td></tr>
<tr><td>1:00 – 2:00 PM</td><td>26.04</td><td>24.06</td><td>Yes</td></tr>
</table></td></tr>
</table>

	<table><tr><td>2:00 – 3:00 PM</td><td>26.29</td><td>24.06</td><td>Yes</td></tr><tr><td>3:00 – 4:00 PM</td><td>26.30</td><td>24.06</td><td>Yes</td></tr><tr><td>4:00 – 5:00 PM</td><td>26.07</td><td>24.06</td><td>Yes</td></tr></table><br><table><tr><th>Time</th><th>Indoor $WBGT_{eff}$ (°C)</th><th>WBGTref (°C)</th><th>Indoor $WBGT_{eff}$ > $WBGT_{ref}$</th></tr><tr><td>9:00 – 10:00 AM</td><td>21.01</td><td>24.06</td><td>No</td></tr><tr><td>10:00 – 11:00 AM</td><td>21.77</td><td>24.06</td><td>No</td></tr><tr><td>11:00 – 12:00 PM</td><td>22.54</td><td>24.06</td><td>No</td></tr><tr><td>12:00 – 1:00 PM</td><td>23.29</td><td>24.06</td><td>No</td></tr><tr><td>1:00 – 2:00 PM</td><td>23.92</td><td>24.06</td><td>No</td></tr><tr><td>2:00 – 3:00 PM</td><td>24.34</td><td>24.06</td><td>Yes</td></tr><tr><td>3:00 – 4:00 PM</td><td>24.55</td><td>24.06</td><td>Yes</td></tr><tr><td>4:00 – 5:00 PM</td><td>24.51</td><td>24.06</td><td>Yes</td></tr></table>
Step 5. Indoor heat stress hours adjusted for local heat and cooling sources	The expert panel assumed that “crop and animal producers” are not exposed to local heat sources or local cooling sources. Therefore, the total number of indoor heat stress hours remains unchanged.
Steps 1 to 5 are repeated for each individual work hour to estimate total annual occupational heat stress exposure.	
Step 6. Heat stress hours adjusted for outdoor and indoor work	Across the full year (including weekends), 111 hours exceeded the $WBGT_{ref}$ threshold for outdoor work, and 38 hours for indoor work. “Crop and animal producers” are classified as working 75% of their time outdoor and 25% of their time indoor. Therefore, the total number of heat stress hours is calculated as:<br><br>(111 * 0.75) + (38 * 0.25) = 93 hours
Step 7. Total heat stress hours adjusted for work organization factors	To reflect a typical work schedule of five working days per week and 30 vacation days per year (230 work hours per year), the total number of heat stress hours was adjusted proportionally:<br><br>93 * 230 / 365 = 58 hours

Supplementary Table S4c. Step-by-step example of heat stress hours calculation using the heat JEM (painters and related workers, ISCO 7141).

<table>
<tr><th>Steps</th><th>Description</th></tr>
<tr><td>Step 1. Outdoor and indoor WBGT</td><td>As an example, the table below presents indoor and outdoor WBGT values (°C) for standard work hours (9:00 AM to 5:00 PM) on 1 August 2020 in France (NUTS 0 level).

<table>
<tr><th>Time</th><th>Outdoor WBGT (°C)</th><th>Indoor WBGT (°C)</th></tr>
<tr><td>9:00 – 10:00 AM</td><td>21.20</td><td>20.01</td></tr>
<tr><td>10:00 – 11:00 AM</td><td>22.52</td><td>20.77</td></tr>
<tr><td>11:00 – 12:00 PM</td><td>23.59</td><td>21.54</td></tr>
<tr><td>12:00 – 1:00 PM</td><td>24.50</td><td>22.29</td></tr>
<tr><td>1:00 – 2:00 PM</td><td>25.04</td><td>22.92</td></tr>
<tr><td>2:00 – 3:00 PM</td><td>25.29</td><td>23.34</td></tr>
<tr><td>3:00 – 4:00 PM</td><td>25.30</td><td>23.55</td></tr>
<tr><td>4:00 – 5:00 PM</td><td>25.07</td><td>23.51</td></tr>
</table>
</td></tr>
<tr><td>Step 2. Outdoor and indoor $WBGT_{eff}$</td><td>For “painters and related workers”, the expert panel assumed no mandatory work clothing or head covering is worn, meaning that WBGT values were taken as $WBGT_{eff}$ values.

<table>
<tr><th>Time</th><th>Outdoor $WBGT_{eff}$ (°C)</th><th>Indoor $WBGT_{eff}$ (°C)</th></tr>
<tr><td>9:00 – 10:00 AM</td><td>21.20</td><td>20.01</td></tr>
<tr><td>10:00 – 11:00 AM</td><td>22.52</td><td>20.77</td></tr>
<tr><td>11:00 – 12:00 PM</td><td>23.59</td><td>21.54</td></tr>
<tr><td>12:00 – 1:00 PM</td><td>24.50</td><td>22.29</td></tr>
<tr><td>1:00 – 2:00 PM</td><td>25.04</td><td>22.92</td></tr>
<tr><td>2:00 – 3:00 PM</td><td>25.29</td><td>23.34</td></tr>
<tr><td>3:00 – 4:00 PM</td><td>25.30</td><td>23.55</td></tr>
<tr><td>4:00 – 5:00 PM</td><td>25.07</td><td>23.51</td></tr>
</table>
</td></tr>
<tr><td>Step 3. $WBGT_{ref}$</td><td>The activity level of “painters and related workers” corresponds to 2.94 metabolic equivalents (METs). The metabolic rate was calculated as:
Metabolic rate = MET * body surface area * 58.2

Using a body surface area of 1.9 m^2, this yields:
2.94 * 1.9 * 58.2 = 325.1 Watts

The $WBGT_{ref}$ value is than calculated as:
$WBGT_{ref} = 59.9 – 14.1 \times \log_{10}(\text{metabolic rate})$
Which results in:
$WBGT_{ref} = 59.9 – 14.1 \times \log_{10}(325.1) = 24.48$ °C</td></tr>
<tr><td>Step 4. Heat stress hours classification ($WBGT_{eff} > WBGT_{ref}$)</td><td>Each hour is classified as a heat stress hour if $WBGT_{eff} > WBGT_{ref}$:

<table>
<tr><th>Time</th><th>Outdoor $WBGT_{eff}$ (°C)</th><th>WBGTref (°C)</th><th>Outdoor $WBGT_{eff} > WBGT_{ref}$</th></tr>
<tr><td>9:00 – 10:00 AM</td><td>22.20</td><td>24.48</td><td>No</td></tr>
<tr><td>10:00 – 11:00 AM</td><td>23.52</td><td>24.48</td><td>No</td></tr>
<tr><td>11:00 – 12:00 PM</td><td>24.59</td><td>24.48</td><td>Yes</td></tr>
<tr><td>12:00 – 1:00 PM</td><td>25.50</td><td>24.48</td><td>Yes</td></tr>
<tr><td>1:00 – 2:00 PM</td><td>26.04</td><td>24.48</td><td>Yes</td></tr>
</table>
</td></tr>
</table>

	2:00 – 3:00 PM	26.29	24.48	Yes
	3:00 – 4:00 PM	26.30	24.48	Yes
	4:00 – 5:00 PM	26.07	24.48	Yes

Time	Indoor $WBGT_{eff}$ (°C)	WBGTref (°C)	Indoor $WBGT_{eff}$ > $WBGT_{ref}$
9:00 – 10:00 AM	21.01	24.48	No
10:00 – 11:00 AM	21.77	24.48	No
11:00 – 12:00 PM	22.54	24.48	No
12:00 – 1:00 PM	23.29	24.48	No
1:00 – 2:00 PM	23.92	24.48	No
2:00 – 3:00 PM	24.34	24.48	No
3:00 – 4:00 PM	24.55	24.48	Yes
4:00 – 5:00 PM	24.51	24.48	Yes

Step 5. Indoor heat stress hours adjusted for local heat and cooling sources	The expert panel assumed that “painters and related workers” are not exposed to local heat sources or local cooling sources. Therefore, the total number of indoor heat stress hours remains unchanged.
Steps 1 to 5 are repeated for each individual work hour to estimate total annual occupational heat stress exposure.	
Step 6. Heat stress hours adjusted for outdoor and indoor work	Across the full year (including weekends), 95 hours exceeded the $WBGT_{ref}$ threshold for outdoor work, and 29 hours for indoor work. “Painters and related workers” are classified as working 43.75% of their time outdoor and 56.25% of their time indoor. Therefore, the total number of heat stress hours is calculated as:<br><br>(95 * 0. 4375) + (29 * 0. 5625) = 58 hours
Step 7. Total heat stress hours adjusted for work organization factors	To reflect a typical work schedule of five working days per week and 30 vacation days per year (230 work hours per year), the total number of heat stress hours was adjusted proportionally:<br><br>58 * 230 / 365 = 36 hours

Supplementary Table S4d. Step-by-step example of heat stress hours calculation using the heat JEM (building construction labourers, ISCO 9313).

<table>
<tr><td>Steps</td><td>Description</td></tr>
<tr><td>Step 1. Outdoor and indoor WBGT</td><td>As an example, the table below presents indoor and outdoor WBGT values (°C) for standard work hours (9:00 AM to 5:00 PM) on 1 August 2020 in France (NUTS 0 level).

<table>
<tr><th>Time</th><th>Outdoor WBGT (°C)</th><th>Indoor WBGT (°C)</th></tr>
<tr><td>9:00 – 10:00 AM</td><td>21.20</td><td>20.01</td></tr>
<tr><td>10:00 – 11:00 AM</td><td>22.52</td><td>20.77</td></tr>
<tr><td>11:00 – 12:00 PM</td><td>23.59</td><td>21.54</td></tr>
<tr><td>12:00 – 1:00 PM</td><td>24.50</td><td>22.29</td></tr>
<tr><td>1:00 – 2:00 PM</td><td>25.04</td><td>22.92</td></tr>
<tr><td>2:00 – 3:00 PM</td><td>25.29</td><td>23.34</td></tr>
<tr><td>3:00 – 4:00 PM</td><td>25.30</td><td>23.55</td></tr>
<tr><td>4:00 – 5:00 PM</td><td>25.07</td><td>23.51</td></tr>
</table></td></tr>
<tr><td>Step 2. Outdoor and indoor $WBGT_{eff}$</td><td>For “building construction labourers”, the expert panel assumed no mandatory work clothing was worn but that head covering was mandatory, adding a +1 °C CAV to the WBGT to obtain WBGTeff values.

<table>
<tr><th>Time</th><th>Outdoor $WBGT_{eff}$ (°C)</th><th>Indoor $WBGT_{eff}$ (°C)</th></tr>
<tr><td>9:00 – 10:00 AM</td><td>22.20</td><td>21.01</td></tr>
<tr><td>10:00 – 11:00 AM</td><td>23.52</td><td>21.77</td></tr>
<tr><td>11:00 – 12:00 PM</td><td>24.59</td><td>22.54</td></tr>
<tr><td>12:00 – 1:00 PM</td><td>25.50</td><td>23.29</td></tr>
<tr><td>1:00 – 2:00 PM</td><td>26.04</td><td>23.92</td></tr>
<tr><td>2:00 – 3:00 PM</td><td>26.29</td><td>24.34</td></tr>
<tr><td>3:00 – 4:00 PM</td><td>26.30</td><td>24.55</td></tr>
<tr><td>4:00 – 5:00 PM</td><td>26.07</td><td>24.51</td></tr>
</table></td></tr>
<tr><td>Step 3. $WBGT_{ref}$</td><td>The activity level of “building construction labourers” corresponds to 4.90 metabolic equivalents (METs). The metabolic rate was calculated as:
Metabolic rate = MET * body surface area * 58.2

Using a body surface area of 1.9 m^2, this yields:
4.90 * 1.9 * 58.2 = 541.8 Watts

The $WBGT_{ref}$ value is than calculated as:
$WBGT_{ref} = 59.9 – 14.1 \times \log_{10}(\text{metabolic rate})$
Which results in:
$WBGT_{ref} = 59.9 – 14.1 \times \log_{10}(541.8) = 21.35$°C</td></tr>
<tr><td>Step 4. Heat stress hours classification ($WBGT_{eff} > WBGT_{ref}$)</td><td>Each hour is classified as a heat stress hour if $WBGT_{eff} > WBGT_{ref}$:

<table>
<tr><th>Time</th><th>Outdoor $WBGT_{eff}$ (°C)</th><th>WBGTref (°C)</th><th>Outdoor $WBGT_{eff} > WBGT_{ref}$</th></tr>
<tr><td>9:00 – 10:00 AM</td><td>22.20</td><td>21.35</td><td>Yes</td></tr>
<tr><td>10:00 – 11:00 AM</td><td>23.52</td><td>21.35</td><td>Yes</td></tr>
<tr><td>11:00 – 12:00 PM</td><td>24.59</td><td>21.35</td><td>Yes</td></tr>
<tr><td>12:00 – 1:00 PM</td><td>25.50</td><td>21.35</td><td>Yes</td></tr>
<tr><td>1:00 – 2:00 PM</td><td>26.04</td><td>21.35</td><td>Yes</td></tr>
<tr><td>2:00 – 3:00 PM</td><td>26.29</td><td>21.35</td><td>Yes</td></tr>
</table></td></tr>
</table>

	3:00 – 4:00 PM 26.30 21.35 Yes<br>4:00 – 5:00 PM 26.07 21.35 Yes

Time	Indoor $WBGT_{eff}$ (°C)	WBGTref (°C)	Indoor $WBGT_{eff}$ > $WBGT_{ref}$
9:00 – 10:00 AM	21.01	21.35	No
10:00 – 11:00 AM	21.77	21.35	Yes
11:00 – 12:00 PM	22.54	21.35	Yes
12:00 – 1:00 PM	23.29	21.35	Yes
1:00 – 2:00 PM	23.92	21.35	Yes
2:00 – 3:00 PM	24.34	21.35	Yes
3:00 – 4:00 PM	24.55	21.35	Yes
4:00 – 5:00 PM	24.51	21.35	Yes

Step 5. Indoor heat stress hours adjusted for local heat and cooling sources	The expert panel assumed that "building construction labourers" are not exposed to local heat sources or local cooling sources. Therefore, the total number of indoor heat stress hours remains unchanged.
Steps 1 to 5 are repeated for each individual work hour to estimate total annual occupational heat stress exposure.	
Step 6. Heat stress hours adjusted for outdoor and indoor work	Across the full year (including weekends), 454 hours exceeded the $WBGT_{ref}$ threshold for outdoor work, and 238 hours for indoor work. "Building construction labourers" are classified as working fully outdoors. Therefore, the total number of heat stress hours is calculated as:<br><br>(454 * 1.0) + (238 * 0.0) = 454 hours
Step 7. Total heat stress hours adjusted for work organization factors	To reflect a typical work schedule of five working days per week and 30 vacation days per year (230 work hours per year), the total number of heat stress hours was adjusted proportionally:<br><br>454 * 230 / 365 = 286 hours
